# Modelling the influence of climate on malaria occurrence in Chimoio Municipality, Mozambique

**DOI:** 10.1186/s13071-017-2205-6

**Published:** 2017-05-25

**Authors:** João Luís Ferrão, Jorge M. Mendes, Marco Painho

**Affiliations:** 1 Faculdade de Engenharia, Universidade Católica de Moçambique, Chimoio, Mozambique; 2 NOVA Information Management Scholl, Universidade Nova de Lisboa, Lisbon, Portugal

**Keywords:** Modelling, Malaria, Chimoio, Precision public health

## Abstract

**Background:**

Mozambique was recently ranked fifth in the African continent for the number of cases of malaria. In Chimoio municipality cases of malaria are increasing annually, contrary to the decreasing trend in Africa. As malaria transmission is influenced to a large extent by climatic conditions, modelling this relationship can provide useful insights for designing precision health measures for malaria control. There is a scarcity of information on the association between climatic variability and malaria transmission risk in Mozambique in general, and in Chimoio in particular. Therefore, the aim of this study is to model the association between climatic variables and malaria cases on a weekly basis, to help policy makers find adequate measures for malaria control and eradication.

**Methods:**

Time series analysis was conducted using data on weekly climatic variables and weekly malaria cases (counts) in Chimoio municipality, from 2006 to 2014. All data were analysed using SPSS-20, R 3.3.2 and BioEstat 5.0. Cross-correlation analysis, linear processes, namely ARIMA models and regression modelling, were used to develop the final model.

**Results:**

Between 2006 and 2014, 490,561 cases of malaria were recorded in Chimoio. Both malaria and climatic data exhibit weekly and yearly systematic fluctuations. Cross-correlation analysis showed that mean temperature and precipitation present significantly lagged correlations with malaria cases. An ARIMA model (2,1,0) (2,1,1)_52_, and a regression model for a Box-Cox transformed number of malaria cases with lags 1, 2 and 3 of weekly malaria cases and lags 6 and 7 of weekly mean temperature and lags 12 of precipitation were fitted. Although, both produced similar widths for prediction intervals, the last was able to anticipate malaria outbreak more accurately.

**Conclusion:**

The Chimoio climate seems ideal for malaria occurrence. Malaria occurrence peaks during January to March in Chimoio. As the lag effect between climatic events and malaria occurrence is important for the prediction of malaria cases, this can be used for designing public precision health measures. The model can be used for planning specific measures for Chimoio municipality. Prospective and multidisciplinary research involving researchers from different fields is welcomed to improve the effect of climatic factors and other factors in malaria cases.

**Electronic supplementary material:**

The online version of this article (doi:10.1186/s13071-017-2205-6) contains supplementary material, which is available to authorized users.

## Background

Mozambique was recently ranked fifth in Africa for the number of malaria cases [1] and reported over six million cases of malaria in 2015 [2]. Chimoio is the capital of Manica Province in the Centre of Mozambique. It is the fifth-largest city in Mozambique, with an estimated population of 324,816 [3], all of whom are at risk of contracting malaria. Malaria is broadly recognised as endemic in Mozambique, with seasonal peaks during the wet season, between November and March, but predominantly in February. In Chimoio municipality cases of malaria are increasing annually, contrary to the decreasing trend in Africa. A recent study on spatial and temporal malaria prevalence in Chimoio municipality indicated that malaria prevalence in Chimoio is 20.1% between 2006 and 2014, with differences in weekly and yearly malaria occurrence [4].

Several institutions operate in Mozambique to control and prevent malaria such as The Lubombo Spatial Development Initiative (LSDI), The President’s Malaria Initiative (PMI), Programa Nacional de Combate a Malaria (CNPM), Unicef, Centre for Disease Control and Prevention (CDC), Centro de Pesquisa de Malaria, and others. Despite these Mozambican Government initiatives, the number of cases continues to increase annually. Most research projects focus on the clinical aspects of the disease such as chemoprophylaxis, and vaccine development. However, disease eradication should not only involve the medical disciplines, but also health economics, geography and ecology, and the social sciences to design and implement control strategies in real life settings [5].

Many time-series studies and studies of epidemics have been carried out to determine explanatory variables for changes in malaria transmission, but most fail to take climatic factors into account [6]. It is well known that the practice of precision health was enabled by the advent of Global Positioning Systems (GPS) and Global Navigation Satellite Systems (GNSS). The Geographical Information System (GIS) is a powerful tool for the health practitioner and researcher’s due to its ability to incorporate data from different sources to produce new information that permits the creation of maps of spatial variability [7].

Public precision health strategies can support decisions to reduce malaria by optimising resource use [8]. For example, decisions can focus spraying efforts to reduce vector numbers, where to build a water body, and when to drain it.

Malaria transmission is highly influenced by environmental and climatic conditions, but the effects are often not linear. The climate-malaria relation is unlikely to be the same over areas covered by different agro-ecological zones [9], thus resources for control have to be spread in time and space. As mentioned by The Global Fund, 90% of malaria cases are related to environmental factors. The level of prevalence can be predicted based on the established relationships between malaria prevalence and environmental data.

Malaria can be cured in cases where the *Plasmodium* parasite is susceptible to the anti-malaria drug, and it can be prevented using indoor and outdoor spraying, mosquito repellents, and bed nets. For significant reduction and elimination, strong and long-term actions are needed. Daily or weekly variations in the values of weather elements and disease data are often of greater importance in determining the efficiency of a climate-disease model. However, most studies only use monthly data [10, 11].

Mathematical models can describe, explain, or predict disease trends/occurrence, they can test multiple scenarios, combine strategies for intervention, and provide a verifiable prediction on what can be expected from implemented schemes [12]. Models using climate variables can predict malaria risk and transmission, and following up such models with research on climate change may help lay the groundwork for malaria prevention and control in Chimoio municipality. Therefore, the objective of this study was to model the effects of several climatic variables (i.e. maximum, minimum, and mean temperature, relative humidity, precipitation, wind speed, visibility and precipitation) on malaria occurrence in Chimoio municipality, using weekly data to define the role of each variable in malaria occurrence.

## Methods

### Study area and population

Chimoio is a municipality in the central region of Mozambique (-19°6′59″S, 33°28′59″E). The population of Chimoio is currently estimated to be 324,816 [3] within an area of 174 km^2^ at a mean altitude of 750 m. The climate is warm and temperate with dry winters from April to July, hot, dry summers from August to October and hot, humid summers from November to March. The average mean temperature is 18 °C, the minimum average temperature is 13.9 °C, and the maximum average temperature is 24 °C. The annual precipitation average is 1143 mm and the wet period is from November to March. The average annual relative humidity (RH) is 67.4% [13].

### Study subjects

Weekly malaria data from the nine-year period (2006 to 2014) were collected from the district Weekly Epidemiological Bulletin (BES) as described elsewhere [5]. Daily climate variables such as daily mean temperature (T), minimum temperature (Tm), and maximum temperature (TM) (°C), relative humidity (RH) (%), wind speed (W) (km/h), visibility (V) (km) and precipitation (P) (mm) were collected from Chimoio Weather Station and, Tutitiempo weather records from the years 2006 to 2014 [14]. The malaria and climate data are included in Additional file 1.

### Data analysis

Weekly cases of malaria and weekly average values for TM, Tm, T, RH, W, V, and P (week 1 to week 52) were calculated and used to estimate the effect of climatic factors on malaria occurrence. All data from climate and clinical records were checked for missing values. Missing values were replaced by the average of nearest values. ANOVA to test differences between years was performed. The model used was:

1 $$ {Y}_{i j}=\mu +{\tau}_i+{\varepsilon}_{i j} $$

where: *μ* is the grand mean, *τ*_*i*_ are deviations from the grand mean due to the treatment levels and, and *ε*_*ij*_ are the error terms [15].

The modelling strategy followed included: (i) exploring malaria cases and climatic variables data through descriptive statistics; (ii) using a Box-Jenkins approach to time series analysis (including transformation and differentiation for stationarity); (iii) using cross-correlation analysis between climatic variables and malaria cases for identification of climatic variables predictor lags; (iv) regression analysis of malaria cases on a malaria moving average forecast (simple exponential smoothing) and on its lags 1, 2 and 3 and lags 6 and 7 of mean temperature and lag 12 of precipitation; and (v) forecasting at regular intervals of 4 weeks for last 52 weeks (2014) left out of model estimation processes.

All statistical analyses were performed with SPSS-20, R 3.3.2 and BioEstat 5. The R script is included in Additional file 2.

## Results

**Fig. 1:**
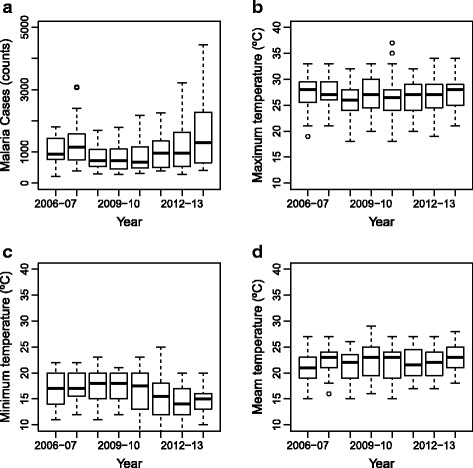
Boxplots of malaria cases (counts) (**a**) and maximum (**b**), minimum (**c**) and mean (**d**) temperature by malaria season (October-September), 2006/2007–2013/2014. Values represent, from top to bottom, maximum, median and minimum

**Fig. 2:**
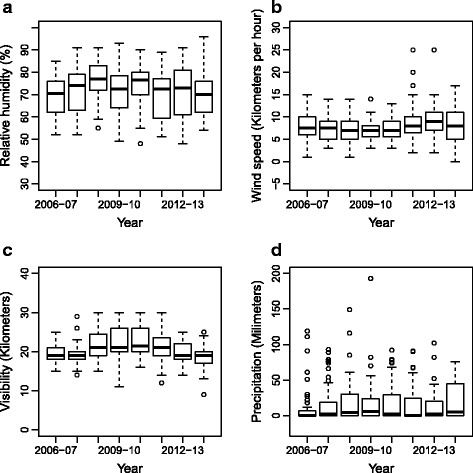
Boxplots of relative humidity (**a**), wind speed (**b**), visibility (**c**) and precipitation (**d**) by malaria season (October-September), 2006/2007–2013/2014. Values represent, from top to bottom, maximum, median and minimum

**Fig. 3:**
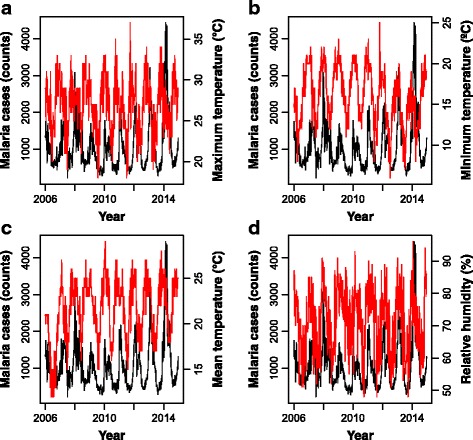
Time series of maximum (**a**), minimum (**b**) and mean temperatures (**c**) and relative humidity (**d**) (right Y-axis). Time series of malaria counts are superimposed in *red* (left Y-axis)

**Fig. 4:**
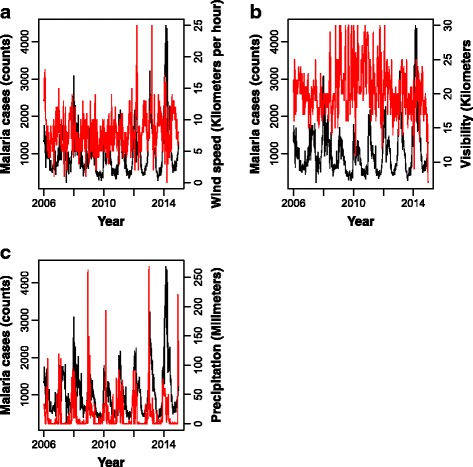
Time series of wind speed (**a**), visibility (**b**) and precipitation (**c**) (right Y-axis). Time series of malaria counts is superimposed in *red* (left Y-axis)

Figures 1 and 2 present box plots of malaria and climate variables for Chimoio by malaria season (October to September 2006 to 2014) along with values for maximum, minimum and the median. Between 2006 and 2014, 490,561 cases of malaria were recorded in Chimoio. The weekly average number of malaria cases was 1048 (SD = 642.12). There were differences in the mean number of cases between malaria season years (*F*_(8,51)_ = 22.1, *P* = 0.0001). Week 40 (in 2006/2007) presented the lowest number of cases, 222, and, week 21 (in 2013/2014) presented the highest number of cases, 4438. The maximum temperature weekly average was 26.9 °C (SD = 3.28), and there were no differences in TM between malaria season years (*F*_(8,51)_ = 2.46, *P* = 0.0132). The minimum temperature weekly average was 16.2 °C (SD = 3.49), and there were significant differences between malaria season years (*F*_(8,51)_ = 20.8, *P* = 0.0001). Mean temperature weekly average was 21.9 °C (SD = 2.92), and there were significant differences between malaria season years (*F*_(8,51)_ = 39.9, *P* = 0.0001). Relative humidity weekly average was 71.7% (SD = 9.86), and there were significant differences between malaria season years in RH (*F*_(8,51)_ = 2.65, *P* = 0.0079). The wind speed weekly average was 7.9 km/h (SD = 3.21), and there were significant differences between malaria season years (*F*_(8, 51)_ = 4.88, *P* = 0.0001). Visibility weekly average was 20.7 km (SD = 43.75), and there were significant differences between malaria season years (*F*_(8,51)_ = 4.88, *P* = 0.0001). Precipitation weekly average was 17.5 mm (SD = 31.95), and there were no differences between malaria season years (*F*_(8,51)_ = 1.5, *P* = 0.144). Annual average precipitation was 913.4 mm (SD = 166.20). Figures 3 and 4 present time series plots of malaria cases (solid black line) and climatic variables (dashed red lines). Both malaria cases series and climatic time series from 2006 to 2014 exhibited seasonal patterns.

**Fig. 5:**
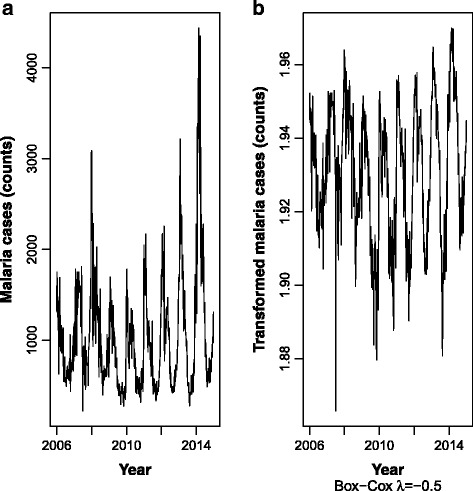
Malaria cases between 2006 and 2014, before (**a**) and after (**b**) Box-Cox transformation (λ = -0.5)

**Fig. 6:**
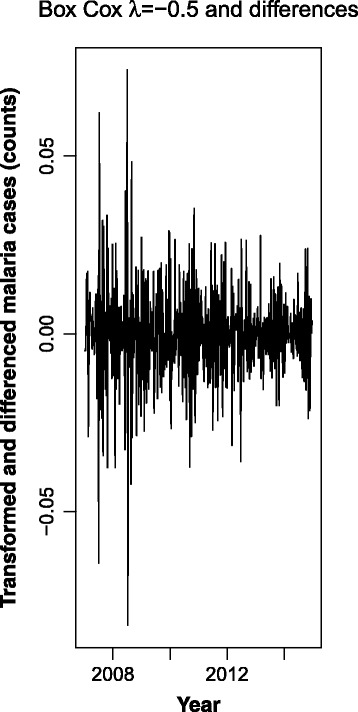
Malaria cases between 2006 and 2014, after Box-Cox transformation (λ = -0.5) and first (lag 1) and seasonal differences (lag 52)

All series presented several peaks and fluctuations. The weekly peaks in the series seem to be separated by more than few weeks indicating a cyclical pattern. Figure 5 presents the time series of malaria cases before and after Box-Cox transformation (λ = -0.5). Figure 5a suggests increasing variability in the malaria cases series along with a slightly increasing trend suggesting both first non-seasonal and seasonal differences might be necessary to turn the series weakly stationary. After applying a Box-Cox transformation (Fig. 5b), the variance was clearly stabilised, and no trend can be overtly observed. Figure 6 presents the time series of malaria cases, between 2006 and 2014, after Box-Cox transformation (λ = -0.5) and non-seasonal first (lag 1) and seasonal differences (lag 52).

**Fig. 7:**
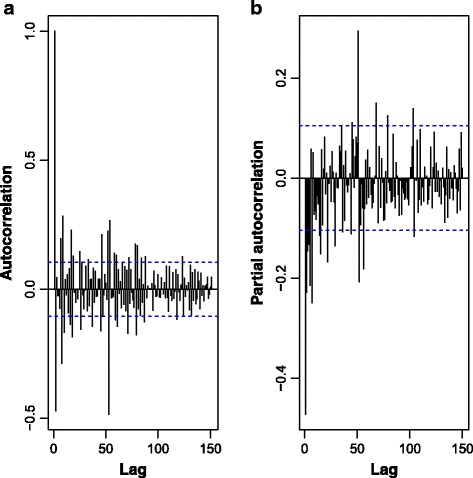
Autocorrelation (**a**) and partial autocorrelation (**b**) functions of the transformed and differenced malaria cases time series in Chimoio, 2006 to 2014. Last 52 weeks, starting at week 1, 2014 (January 2014 through December 2014) were left out for model forecasting assessment. Autocorrelation is plotted up to lag 150

Figure 7 presents the autocorrelation (ACF) partial autocorrelation (PACF) functions of the transformed and differenced malaria cases time series in Chimoio. Autocorrelation is plotted up to lag 150. For modelling purposes, the last 52 weeks, starting at week 1, 2014 (January 2014 through December 2014), were left out for forecasting assessment.

**Fig. 8:**
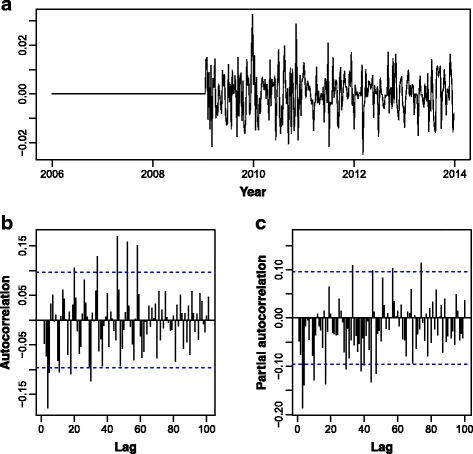
Diagnostic checks of ARIMA (2,1,0) (2,1,1)_52_ residuals. **a** Time series of residuals. **b** Autocorrelation function of residuals. **c** Partial autocorrelation function of residuals

**Fig. 9:**
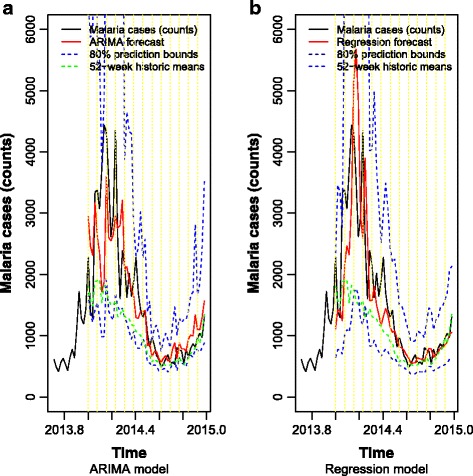
ARIMA (2,1,0) (2,1,1)_52_ (**a**) and regression (**b**) forecasts of the last 52 observations that were left out of the modelling procedure. *Black*, *red* dashed *blue* and *green* lines represent, malaria cases (counts) and their forecasts, 80% confidence limits and 52-week historic means, respectively. Dashed *yellow* vertical lines denote the thirteen 4-week long prediction periods

Both the ACF and PACF suggest ARMA(2,0) and ARMA(2,1) patterns for non-seasonal and seasonal components leading to a Seasonal ARIMA(2,1,0) (2,1,1)_52_. Indeed, among the all experimented models (up to the second order in autoregressive and moving average components) ARIMA (2,1,0) (2,1,1)^52^ was the one leading to the smallest AIC to the Box-Cox transformed series:

2 $$ \left(1-{\phi}_1 B-{\phi}_2{B}^2\right)\kern0.5em \left(1-{\Phi}_1{B}^{52}-{\Phi}_2{B}^{104}\right)\kern0.5em \left(1-{B}^{52}\right)\kern0.5em \left(1- B\right)\kern0.5em {y}_t\kern0.5em =\kern0.5em \left(1+\kern0.5em {\varTheta}_1{B}^{52}\right)\kern0.5em {e}_t $$

where *y”*_t_ is the Box-Cox transformed malaria cases series, *e*_t_ is considered white noise and _*Φ*1_ = -0.3395 (standard error, SE = 0.0518), _*Φ*2_ = -0.2323 (SE = 0.0511), Φ_1_ = -0.4299 (SE = 0.0551), Φ_2_ = -0.2672 (SE = 0.0426), and θ_1_ = -0.3267 (SE = 0.0843). All the coefficients were statistically significant at 0.05. Diagnostic checks for residuals of the estimated model are presented in Fig. 8. Residual autocorrelation was still significant for some lags. Prediction for the last 52 observations (the entire year of 2014, which contains the last malaria outbreak peak) that were left out of the modelling procedure is presented in Fig. 9a. Forecasting was done on a 4-week long period basis (*t + 1*, *t + 2*, *t + 3*, *t + 4*), based on an estimated seasonal ARIMA model for data up to *t*. On the one hand, a 4-week long forecasting period is not large enough to produce inaccurate forecasts by a seasonal ARIMA model. On the other hand, it is sufficiently large to anticipate perfectly manageable Precision Public Health malaria outbreak evolution. Figure 9b, besides the data, mean forecasts and respective 80% confidence prediction limits, also contains the historical means of weeks 1 to 52 (dashed yellow vertical lines), which given the raising pattern of malaria cases in the last years tends to underestimate the outbreak peak. Although the last weeks’ forecasts followed the actual values, it seems to be underestimating the outbreak peak.

Although the purpose of this first modelling step was to approximate a possible model for malaria cases time series, the goal of this study was to find a prediction model for malaria cases that can take advantage of the relationship between malaria and climatic variables.

**Fig. 10:**
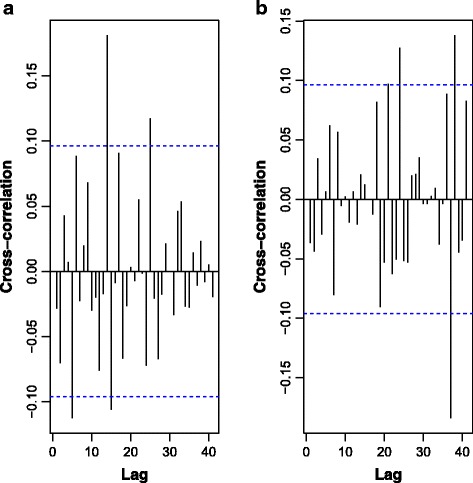
Cross-correlation functions of Box-Cox-transformed and differenced (first and seasonal differences) of minimum temperature (**a)** and precipitation (**b**)

**Fig. 11:**
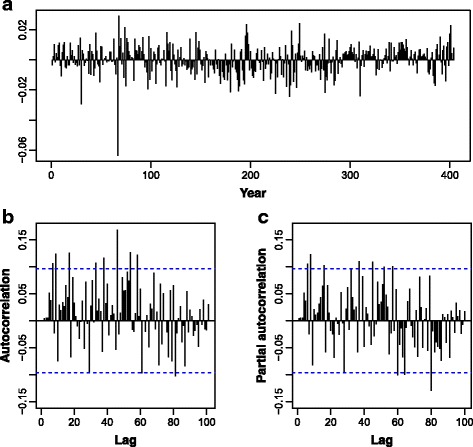
Diagnostic checks of regression model (3) residuals (coefficient of determination *R* ^2^ = 72.5. **a** Time series of residuals. **b** Autocorrelation function of residuals. **c** Partial autocorrelation function of residuals

A cross-correlation analysis was performed to find the best predictor lags of the climatic variables. To keep interpretability, first and seasonal differences (lag 52) were applied to climatic variables prior to cross-correlation calculation. No stabilising variance transformation was applied as the predictors will be used in a regression setting where the predictors are assumed non-stochastic variables. Several climatic variables exhibited significant cross-correlations with malaria past (negative) lag 52. Therefore, attention was drawn to (negative) lags that are known to be closely related to parasite life cycle, namely lags -1, -2, …, 12 weeks. Only minimum temperature (lags -6 and -7) and precipitation (lag -12) exhibited significant cross-correlation. Figure 10 presents the cross-correlation functions between minimum temperature and precipitation (after first and seasonal differences) and Box-Cox transformed and differenced malaria cases. Lags -1, -2, and -3 of the Box-Cox transformed malaria cases series and lags -6, and -7 of minimum temperature and lag -12 of precipitation were used in the regression model. Historical means of malaria cases (already discussed) could provide insightful information for the regression model. To introduce in the regression model the memory of the time series process, it was decided to include in the regression model, as an independent variable, one-step-ahead forecasts of a simple exponential smoothing model (α = 0.6). The estimated model is:

3 $$ {y}_{{}_t}^{*}\kern0.5em =\kern0.5em {\beta}_0+{\beta}_1{y_{t-1}^{ses}}_{\left| t\right.}+{\beta}_2{y}_{t-1}^{*}+{\beta}_3{y}_{t-2}^{*}+{\beta}_4{y}_{t-3}^{*}+{\beta}_5 T{m}_{t-6}+{\beta}_6 T{m}_{t-7}+{\beta}_7{P}_{t-12}+{\eta}_t, $$

where *y* ^*^_t_ denotes for Box-Cox transformed malaria cases series (with no differences), *y* ^*ses*^ is the simple exponential smoothing forecast at *t*, using data up to *t-1* (α = 0.6) and *Tm* and *P* correspond to minimum temperature and precipitation time series. The regression coefficients *β*_0_ = 0.0701 (SE = 0.0152), *β*_2_ = 0.0370 (SE = 0.0065), *β*_3_ = 0.0194 (SE = 0.0056) and *β*_6_ = 0.0008 (SE = 0.0003) were marginally statistically significant at *P* = 0.05 and *R* ^2^ = 0.726. Residual analysis shows the model can capture almost all temporal dependence, as despite some autocorrelations being statistically significant, they are smaller than 0.2 (Fig. 11).

To compare with previous ARIMA model, in the estimation process, the last 52 observations were left out for forecast assessment purposes. Figure 9b presents last 52 point forecasts of the regression model (3) along with the 95% confidence limits in the original scale, i.e. after applying inverse Box-Cox transformation (λ = −0.5). Forecasts were done on 4-week forecast bases as before. Point’s forecasts seem to follow closely malaria series values, though the outbreak peak is being overestimated. The width of prediction intervals was like the ones produced by seasonal ARIMA model (close to 600 cases, an accuracy perfectly manageable by Public Precision health), though anticipation of outbreak’s peak seems to be more accurate (last observations of 2014).

## Discussion

Although malaria shows seasonality according to the climate, very few studies have been conducted on the association between the malaria occurrences with climate variables using weekly resolution and with high malaria occurrence volume in the Southern region of Africa, giving more accurate results.

In this study, malaria cases are increasing, contrary to the decreasing tendency reported in neighbouring Malawi [16], and, South Africa [17]. This could be probably due to improved accessibility to health centres and decreased vector control due to the scarcity of resources for malaria control.

On average, week 6 presented the peak of malaria cases and week 33 the lowest number of cases of malaria; these results are consistent with previously published studies in Mozambique, Maputo [18], and Chimoio [4]. The ARIMA model developed in this study, ARIMA (2,1,0) (2,1,1)_52_, attempted to provide an easy technique to predict the expected number of malaria cases per week based on past observed cases, although it does not account for climate factors.

Cross-correlation analysis showed that mean temperature, and precipitation presented significantly lagged correlations with malaria cases. A regression model of a differenced (lag1 and lag 12) Box-Cox transformation (λ = -0.5) of malaria cases on lag 1, 2 and 3 of weekly malaria cases and lag 6 and 7 of weekly mean temperature and lag 12 of precipitation was found as the best prediction model for weekly malaria cases.

As shown in Fig. 9, historical means failed completely, especially at the peak of the malaria occurrence. Although the two models developed in this study produced prediction intervals having widths of some hundred cases, the regression model was the one able to anticipate accurately the peak of the occurrence. ARIMA model was also used for malaria forecasting in South Africa [17], Zambia [19], Burundi [20] and India [21] with comparable results.

Malaria transmission occurs throughout the year with peaks between weeks 1 to 12. The onset of rain occurs in mid-November. This indicates that malaria occurrence has a strong association with rainfall six to eight weeks before, coinciding, with the malaria cycle three components: (i) the growth of the *Anopheles* female mosquito from egg to adult to parasite transmission; (ii) the development of the *Plasmodium* parasites (gametocyte to sporozoites) that are able to infect humans; and (iii) the incubation period in the human host from infection to malaria symptoms [22, 23]. Thus malaria occurrence peak can be expected 45 to 60 days after the onset of rain. Similar results were also found in Mozambique [4] and South Africa [17]. Increased precipitation can provide more breeding sites for mosquitoes, but excess rain can also destroy breeding sites [24].

Temperature affects the development of malaria; the parasite does not develop below 18 °C and over 40 °C [25, 26]. A rise in temperature can reduce the time for production of new generations and also shortens the incubation period of the parasite in mosquitoes. Sporogonic cycles take about 9 to 10 days at temperatures of 28 °C, but temperatures above 30 °C and below 16 °C have a negative impact on parasite development [27]. The highest proportion of vectors surviving the incubation period is observed at temperatures between 28 and 32 °C [28]. In this study, the average maximum temperature recorded was 26.8 °C ranging between 22.3 and 31 °C suggesting that Chimoio is the ideal location for malaria breeding. Minimum temperature in the present study was below 18 °C from week 10 to 40, coinciding with an accentuated reduction in malaria occurrence. In this study, the mean temperature was found to be a significant predictor for malaria occurrence, similar to studies carried out in South Africa [17] and Burundi [20].

Relative humidity (RH) also plays a role in malaria episodes, and mosquitoes become more active when humidity rises. If the average monthly relative humidity is below 60%, it is believed that the life of the mosquito is so short that very little or no malaria transmission is possible [29, 30]. Relative humidity in this study was 72.1% and only four weeks of the year presented RH less than 60% implying that humidity does not restrict malaria occurrence in Chimoio. Similar results were also reported in a study in Ghana [23].

Wind speed was found to be a significant influence in malaria occurrence in Nigeria [11, 26]. In this study, the wind speed was not found to be a significant predictor for malaria occurrence in Chimoio. Visibility was not found to be a significant predictor for malaria occurrence consistent in studies in Nigeria [11] and South Africa [17]. Most *Anopheles* mosquitoes are crepuscular (active at dusk or dawn) or nocturnal (active at night) [31].

It was found that fog day frequency had a positive effect on malaria incidence in the following year [32].

The *R*-square in this study was 0.725 implying that 72.5% of the variance in malaria occurrence can be explained by variance in the predictive variables. In Burundi, 82% was reported [20]. The results are higher than a study in Nigeria that found 66% [15] and lower than the Global Fund Report [1] that indicated that 90% of malaria cases are related to environmental factors. Other factors such as poor prevention strategies, lack of funds, poor sanitation, inadequate drainage systems, and planning problems, amongst others, also contribute to the occurrence of malaria. Geographical and environmental factors such as altitude and land cover are also variables that influence malaria occurrence [33]

The assumption the factors other than climate remained constant over the period, is a limitation of the present model that makes it difficult to generalize the results to other regions. From the results of the present study, it can be stated that malaria occurrence in Chimoio depends on to a large extent on precipitation, and mean temperature. The results also indicate that if strong actions are not taken at the right time and place, malaria cases will continue to occur in the municipality.

This model is robust and, can predict the expected number of malaria cases 3.5 months in advance and, timely prevention and control measures can be effectively planned in Chimoio, such as the elimination of vector breeding places, correct time and place to spray insecticides, and awareness campaigns weeks before the malaria peak season. This can lead to a reduction in malaria cases, by knowing the best moment for spraying, saving time and cost of insecticide application and, preventive programmes, and guiding smart environmental care.

## Conclusion

The Chimoio climate seems ideal for malaria occurrence. A seasonal pattern was observed in malaria occurrence in Chimoio with peaks during weeks 1 to 12 (January to March). Since the lag effect between climatic events and malaria occurrence is important for malaria cases prediction this can be used for designing Precision Public Health measures. The model can be used for planning specific measures for Chimoio municipality. The results from this study cannot confirm or rule out a prediction for areas with similar altitude and precipitation as Chimoio. Prospective and multidisciplinary research involving researchers from different fields is welcomed to improve the effect of climatic factors and other factors in malaria cases. The model can also be applied to analyse the spread of other infectious diseases and in optimising management efforts.

## Additional files

Additional file 1: Data.**Table S1.** Chimoio malaria and climate data from 2008 to 2014. **Table S2.** Maximum temperature for Chimoio 2008 to 2014. **Table S3.** Minimum temperature for Chimoio 2008 to 2014. **Table S4.** Mean temperature for Chimoio 2008 to 2014. **Table S5.** Relative humidity for Chimoio 2008 to 2014. **Table S6.** Wind speed for Chimoio 2008 to 2014. **Table S7.** Visibility for Chimoio 2008 to 2014**. Table S8.** Minimum temperature for Chimoio 2008 to 2014. **Table S9.** Malaria in Chimoio 2008 to 2014. (XLSX 189 kb)

Additional file 2: R script. (TXT 6 kb)
